# A heart in a heart: a case report of spontaneous flail of bicuspid aortic valve

**DOI:** 10.1186/s13256-021-03211-8

**Published:** 2021-12-28

**Authors:** Khadije Mohammadi, Mahsa Akrami, Marzieh Mirtajaddini

**Affiliations:** 1 Cardiovascular Research Center, Shafa Hospital, Kerman University of Medical Sciences, Shafa Avenue, Kerman, Iran; 2 Present Address: Rajaie Cardiovascular Medical and Research Center, Iran University of Medical Sciences, Tehran, Iran

**Keywords:** Bicuspid aortic valve, Aortic regurgitation, Aortic valve flail, Case report

## Abstract

**Background:**

Aortic regurgitation is attributed to congenital and acquired abnormalities of the aortic valve or aortic valve supporting structures. The most common cause of aortic regurgitation is atherosclerotic degeneration of the valve, especially in the presence of a bicuspid aortic valve.

**Case summary:**

A 25-year-old Persian man with no past medical history, developed dyspnea since 1 week before his first visit to the physician. He was an active person up to this time, and had no history of trauma or chest pain. Transthoracic echocardiography showed severe aortic regurgitation in the context of flail bicuspid aortic valve, with no evidence of endocarditis. Laboratory tests including blood cultures were negative for infection. The patient underwent aortic valve replacement and the diagnosis was confirmed at time of surgery.

**Conclusion:**

This case represents noninfective and nontraumatic spontaneous flail of bicuspid aortic valve.

**Supplementary Information:**

The online version contains supplementary material available at 10.1186/s13256-021-03211-8.

## Introduction

Aortic regurgitation (AR) is related to diverse congenital and acquired abnormalities of the aortic valve (AV) or AV supporting structures. The most common cause of AR is atherosclerotic degeneration of the valve, especially in the presence of a bicuspid aortic valve (BAV). BAV is a common congenital abnormality seen in 0.5–2% of the general population and predisposes to aortic valve stenosis and regurgitation [1].

The flail of aortic valve in the context of BAV is a rare clinical entity that usually causes severe AR; AV flail usually originates from destruction of aortic cusps or supporting structures as in bacterial endocarditis, or may be due to traumatic injury or redundant valve cusps resulting from myxomatous degeneration of the valve [2].

Here, we report a case of spontaneous flail of bicuspid aortic valve that resulted in severe AR without any obvious cause.

## Case report

A 25-year-old Persian man was referred to our center with complaint of dyspnea on exertion since 1 week ago. COVID-19 infection was considered and rejected in another center by a negative nasopharyngeal swab polymerase chain reaction (PCR) test. Because of a cardiac murmur in his examination, he was referred to us. The patient had no past medical history but his father had undergone aortic valve replacement (AVR) surgery because of congenital aortic valve (AV) disease.

He had no history of fever, cough, chest pain, orthopnoea, chest wall trauma, or heavy exercise. His hemodynamic was stable, no fever was detected, and there was no respiratory distress. In physical examination, a diastolic murmur in aortic foci was heard. Lung sounds were clear and there was no peripheral edema.

**Fig. 1:**
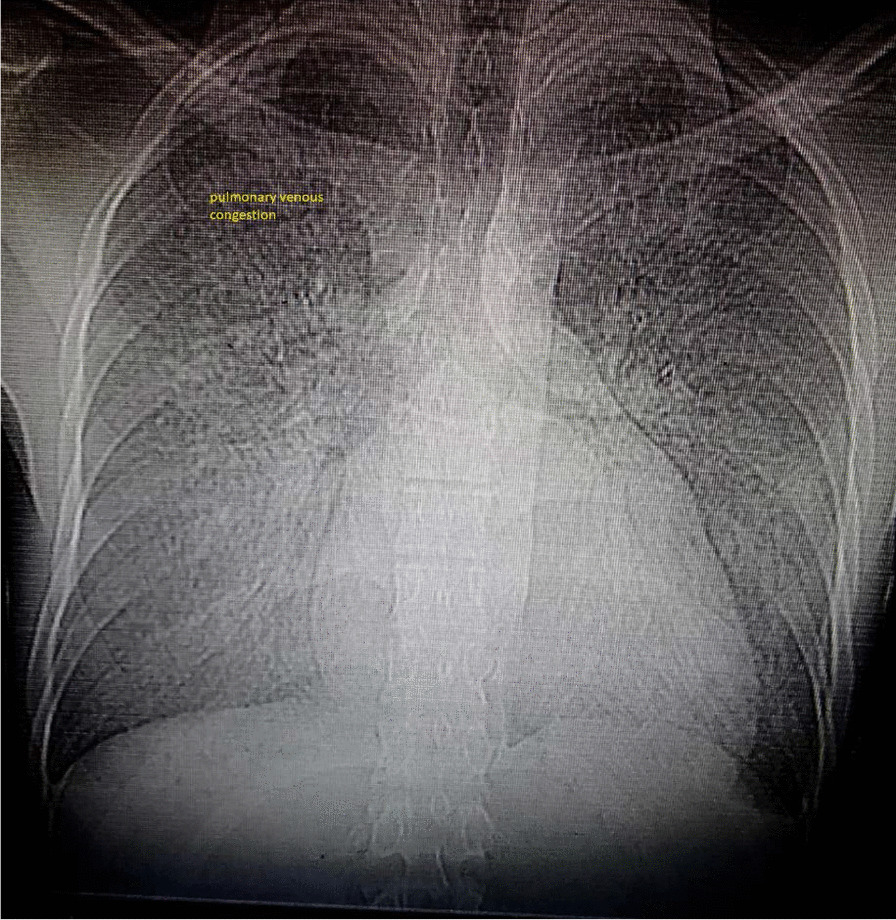
Chest X-ray showed increased cardiothoracic ratio with pulmonary venous congestion

Chest X-ray (CXR) revealed increased cardiothoracic ratio and pulmonary vascular marking compatible with interstitial pulmonary edema. (Fig. 1)

In laboratory tests, erythrocyte sedimentation rate (ESR) was normal and C-reactive protein (CRP), procalcitonin, and blood culture were negative.

**Fig. 2:**
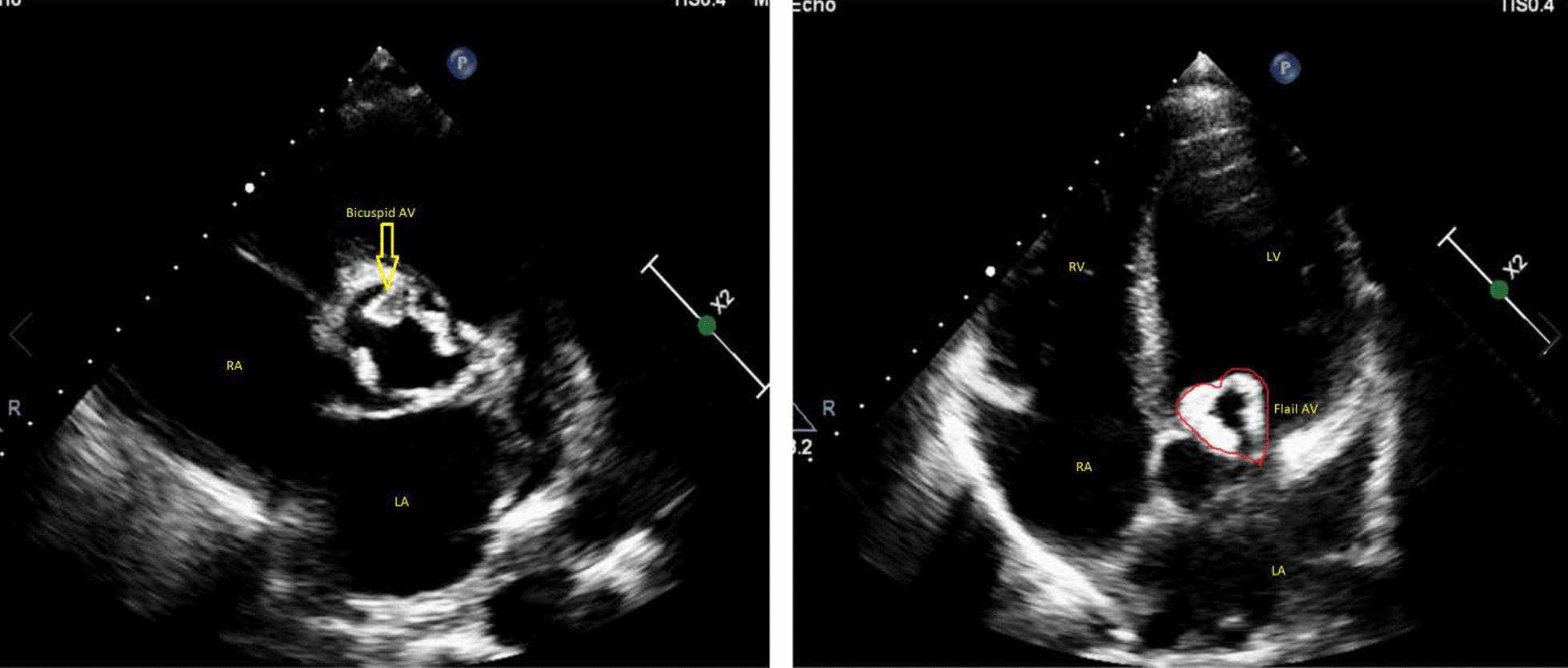
Left panel: Parasternal short axis view demonstrated thickened, calcified, and bicuspid aortic valve (arrow). Right panel: Apical five-chamber view showed flail of AV that mimics a heart configuration.

Transthoracic echocardiography revealed mild left ventricular (LV) enlargement (LV end diastolic volume index :82 cc/m^2^) and mild systolic dysfunction (LV ejection fraction 50%), increased LV filling pressure (E/e’ :20) and mild-to-moderate left atrium (LA) enlargement (LAVI :42 cc/m^2^). Mitral, tricuspid, and pulmonary valves were normal without significant regurgitation or stenosis. However, premature closure of mitral valve was detected. Aortic valve was thickened, calcified, and bicuspid (Additional file 1: Video S1), with flail of both leaflets that mimics a heart configuration in apical five-chamber view (Fig. 2, Additional file 2: Video S2, Additional file 3: Video S3) and resulted in free aortic regurgitation (AR). No vegetation, abscess, or any evidence of infective endocarditis was detected by transesophageal echocardiography and no significant aortic root abnormality was seen. These findings were interpreted as an acute flail of AV in the context of previous chronic AR, and we tried to rule out causes of acute AR. The patients had no history of trauma, no evidence of infective endocarditis was found in his workup, and no dissection was detected on imaging.

After treatment with diuretics (because of elevated LV filling pressure), the patient underwent mechanical aortic valve replacement surgery. Intraoperative observation reported ruptured bicuspid aortic valve with no vegetation or abscess formation. Pathologic evaluation of valve tissue revealed hyalinization and calcification, and no evidence of infection was found in pathology and culture.

After successful heart surgery and patient recovery, he was discharged home and 6- and 12-month follow-ups were uneventful.

## Discussion

In bicuspid aortic valve, aortic incompetence is relatively common. The mechanisms are prolapsing cusps, endocarditis, dilation of ascending aorta, aortic dissection, and, importantly, myxoid degeneration [3]. Although the clinical course is usually slowly progressive, in some cases acute AR can occur and is a life-threatening condition. The common causes of acute AR are aortic root dissection, infective endocarditis, and thoracic trauma. However, other infrequent causes have been reported. Flail of aortic valve due to spontaneous aortic laceration [4] or high-voltage electrical injury [5] have been reported as infrequent causes of acute AR in two case reports. Spontaneous localized intimal tear of ascending aorta [6], rupture of an aortic commissure [7], and spontaneous rupture of a bicuspid aortic valve following extensive exercise [8] have also been reported as rare causes of acute AR.

Fenestration of aortic valve is a common condition that rarely cause aortic incompetency. In one study, it accounts for 3.1% of AR mechanisms among patients [9].Usually, fenestrations are located in the commissures, and their frequency is higher in men, BAV, and valves with myxomatous degeneration. Regurgitation can occur in large fenestration or, in rare cases, in spontaneous rupture of fenestration [10].

A subgroup of patients with BAV and AR not attributed to infection have an anomalous cord extending from the raphe of one of the two cusps to the wall of the aorta, which keeps the cusp from prolapsing toward the left ventricle. In patients who presented with acute AR, the cord had ruptured and resulted in flail of the cusp toward the left ventricle cavity and the acute onset of symptoms [11].

## Conclusion

In this case, we could not find any particular cause of AV flail and severe AR. In our evaluation, there was no evidence of infective endocarditis or aortic root dissection, and the patient did not have a history of chest wall trauma or heavy exercise. No fenestration or ruptured strand was found at the time of surgery. Calcification and hyalinization were reported in pathologic evaluation and no evidence of infection was seen. Therefore, we report this case as a nontraumatic and noninfective spontaneous flail of bicuspid AV.

## Supplementary Information

**Additional file 1.** Parasternal short axis view of transthoracic echocardiography that showed thickened, calcified and bicuspid aortic valve.

**Additional file 2.** Parasternal long axis view of transthoracic echocardiography that showed flail of aprtic valve.

**Additional file 3.** Apical five chamber view of echocardiography that demonstrated heart configuration of flail aortic valve.

## Data Availability

The datasets used in the study are available from the corresponding author.
